# Platelet and Neutrophil Responses to Gram Positive Pathogens in Patients with Bacteremic Infection

**DOI:** 10.1371/journal.pone.0026928

**Published:** 2011-11-29

**Authors:** Daniel Johansson, Oonagh Shannon, Magnus Rasmussen

**Affiliations:** Division of Infection Medicine, Department of Clinical Sciences, Lund University, Lund, Sweden; Charité-University Medicine Berlin, Germany

## Abstract

**Background:**

Many Gram-positive pathogens aggregate and activate platelets *in vitro* and this has been proposed to contribute to virulence. Platelets can also form complexes with neutrophils but little is however known about platelet and platelet-neutrophil responses in bacterial infection.

**Methodology/Principal Findings:**

We added isolates of Gram-positive bacteria from 38 patients with a bacteremic infection to blood drawn from the same patient. Aggregometry and flow cytometry were used to assess platelet aggregation and to quantify activation of platelets, neutrophils, and platelet-neutrophils complexes (PNCs) induced by the bacteria. Fifteen healthy persons served as controls. Most isolates of *Staphylococcus aureus*, beta hemolytic streptococci, and *Enterococcus faecalis* induced aggregation of platelets from their respective hosts, whereas pneumococci failed to do so. *S. aureus* isolates induced platelet aggregation more rapidly in patients than in controls, whereas platelet activation by *S. aureus* was lower in patients than in controls. PNCs were more abundant in baseline samples from patients than in healthy controls and most bacterial isolates induced additional PNC formation and neutrophil activation.

**Conclusion/Significance:**

We have demonstrated for the first time that bacteria isolated from patients with Gram-positive bacteremia can induce platelet activation and aggregation, PNC formation, and neutrophil activation in the same infected host. This underlines the significance of these interactions during infection, which could be a target for future therapies in sepsis.

## Introduction

Sepsis remains a major cause of mortality, causing over 200 000 deaths annually in the US alone [1]. Although substantial advances have been made in the understanding of this disease [2], these new insights have not yet translated into successful therapeutic strategies [3].

Sepsis is the consequence of a complex host response to an invading microorganism [4]. Invading bacteria are recognized by innate immune cells leading to the release of multiple cytokines which in concert with the activation of other defense systems can result in the clinical picture known as systemic inflammatory response syndrome (SIRS).

While most of the attention in the sepsis field has been devoted to traditional immune cells, there is mounting evidence that platelets are essential not only in coagulation but that they are also important in inflammation and defense against infection [5]. Most of the Gram-positive pathogens that commonly cause sepsis, including *Staphylococcus aureus*, beta hemolytic group A streptococci (GAS), as well as several other streptococcal species and *Enterococcus faecalis* are known to activate and aggregate human platelets [6], [7], [8], [9]. The molecular mechanisms leading to platelet activation has been described in detail for many bacterial species, and it is clear that host factors such as specific Immunoglobulin G (IgG) play an important role [10], [11], [12], [13], [14], [15]. In support of a role for platelets in defense against Gram-positive bacterial infection, thrombocytopenia in patients with *S. aureus* bacteremia is associated with poor prognosis [16].

Platelets and polymorphonuclear granulocytes are known to adhere to each other and form platelet-neutrophil complexes (PNCs). Platelet P-selectin and the Mac-1 complex of neutrophils have been shown to be the main regulators in the formation of PNCs [17], [18], [19], PNCs have been proposed to play a role in the development of multi-organ failure in severe disease due to increased sequestration in liver sinusoids and pulmonary capillaries [20], as well as in promoting neutrophil activation and migration, and in the formation of neutrophil extracellular traps which can ensnare bacteria [21].

Despite evidence that there are important links between platelets, bacteria and leukocytes, the specific role of these interactions during infection has yet to be elucidated. Relatively few studies have been carried out on host platelet and neutrophil responses to specific pathogens: Peters et al. [22] investigated platelet-neutrophil interactions in severe meningococcemia and found that it was associated with neutrophil but not platelet activation and suggested that the PNC formation seen in this disease was potentiated by neutrophil activation. Nicu et al. [23] compared platelet and leukocyte response to oral bacteria in patients with periodontitis to that in healthy controls and found that all bacteria tested induced PNC formation.

Collectively, the data from *in vitro* studies imply that platelet-bacteria interactions may be important during infection. In this study we have enrolled 38 patients with Gram-positive bacteremia and we studied platelet-neutrophil interactions in patient blood in response to the invading bacterium.

## Results

### Patients and isolates

Patients were included from February of 2009 through May of 2010. All 38 patients included in the study were judged to have an infection by the responsible physician. Patient characteristics are shown in Table 1, where they are grouped according to isolate. Sixteen patients were female and 22 were male. The mean age was 67 years (range, 23–98). Seven patients had diabetes mellitus, 6 had chronic obstructive lung disease, and 4 had congestive heart failure. Nine patients did not have any apparent underlying disorder. The most common bacterium isolated was *S. aureus* (n = 13), followed by *S. pneumoniae* (n = 11), GAS (n = 4) and beta-hemolytic group G streptococci (GGS) (n = 2), enterococci (n = 5), alpha-hemolytic streptococci (n = 3), and group B streptococci (GBS) (n = 1). Given the many similarities between the beta-hemolytic streptococci (BHS) [24], these bacteria were considered as one entity in all statistical analyses.

**Table 1:** Patient characteristics.

Patient	Gender (Age)	Bacterium	Focal infection	Underlying disorders	Antiplatelet medication	SIRS signs on day of admittance	SIRS signs on day of experiment
1	F (76)	*S. aureus*	Cutaneous carbuncle	DM (type II)	-	2/4	0/4
2	M (77)	*S. aureus*	Pneumonia	COPD, CHF	A, C	2/4	2/4
3	M (72)	*S. aureus*	Septic arthritis	COPD, vasculitis	-	2/4	2/4
4	M (73)	*S. aureus*	Cellulitis	Colon cancer	-	0/3	0/4
5	M (39)	*S. aureus*	Unknown	Nephrostomy, Colostomy	-	1/3	1/4
6	M (23)	*S. aureus*	IE	Dermatitis	-	2/4	0/3
7	F (65)	*S. aureus*	Pneumonia	DM (type II)	A	1/1	1/4
8	M (91)	*S. aureus*	In diabetic leg ulcers	DM (type II)	A	2/4	1/4
9	F (42)	*S. aureus*	Pacemaker endocarditis	Pacemaker	-	1/3	0/4
10	M (77)	*S. aureus*	Unknown	CHF	A, C	1/3	0/4
11	F (98)	*S. aureus*	IE	Nasal cavity cancer, CHF	-	3/3	1/4
12	M (59)	*S. aureus*	Unknown	CMT	-	2/3	1/4
13	F (84)	*S. aureus*	IE	Pacemaker, CHF, PBC	-	2/3	0/4
14	M (71)	*S. pneumoniae*	Septic arthritis	DM (type I)	A	2/4	0/1
15	M (49)	*S. pneumoniae*	Pneumonia	None	-	4/4	3/4
16	F (65)	*S. pneumoniae*	Pneumonia	COPD	I	0/1	1/4
17	M (87)	*S. pneumoniae*	Pneumonia	None	A	1/4	0/4
18	F (58)	*S. pneumoniae*	Pneumonia	COPD, Asthma	-	2/4	1/3
19	F (74)	*S. pneumoniae*	Pneumonia	COPD	-	2/4	0/4
20	F (42)	*S. pneumoniae*	Pneumonia	None	-	1/4	4/4
21	M (39)	*S. pneumoniae*	Acute sinusitis	None	-	1/4	0/4
22	F (57)	*S. pneumoniae*	Unknown	None	-	2/4	3/4
23	F (67)	*S. pneumoniae*	Pneumonia	DM (type II)	A	2/3	2/4
24	F (64)	GAS	Septic arthritis	None	-	2/3	2/4
25	M (84)	GGS	In necrotic foot ulcers	Necrotic foot ulcers	-	1/4	1/4
26	M (88)	GGS	In chronic leg ulcers	Chronic leg ulcers	-	2/4	2/4
27	F (40)	GAS	Para-pharyngeal abscesses	None	-	2/3	2/4
28	M (75)	GAS	Wound infection	DM (type II), Urinary bladder cancer	-	2/4	0/4
29	M (75)	GAS	Septic arthritis	None	-	3/4	2/3
30	M (80)	*E. faecalis*	Unknown	DM (type II)	A	2/2	0/4
31	M (74)	*E. faecalis*	IE	Prosthetic heart valve	A	2/4	0/4
32	M (84)	*E. faecalis*	Unknown	Long-term indwelling catheter, COPD	A	1/4	1/4
33	M (73)	*E. faecalis*	Pyelitis	Prostatic hypertrophy	A	4/4	1/4
34	F (71)	*E. faecium*	Post-surgical abdominal infection	Gastro-intestinal carcinoid	A, I	2/2	0/4
35	M (67)	*S. mutans*	IE, Infectious spondylitis	None	-	0/4	0/4
36	F (74)	*S. gordonii*	IE	Prosthetic heart valve	-	2/4	0/4
37	M (48)	*S. mitis*	IE	Mitral insufficiency	-	0/4	1/4
38	F (74)	GBS	Erysipelas	Lymphedema	-	2/4	0/4

Abbreviations: M = Male. F = Female. SIRS = systemic inflammation response syndrome. Numbers are given as the parameters fulfilled by the number parameters registered. For definitions, see the text.

IE = Infectious endocarditis. DM = Diabetes mellitus. COPD = Chronic Obstructive Pulmonary Disease. CHF = Congestive heart failure. CMT = Charcot-Marie-Tooth Disease. PBC = Primary biliary cirrhosis.

GAS = Group A streptococcus. GGS = Group G streptococcus. GBS = Group B streptococcus. A = Aspirin. C = Clopidogrel. I = Ibuprofen.

The most common focal infection was pneumonia (n = 9) followed by infectious endocarditis (IE) (n = 8, of which one case was pacemaker endocarditis). Seven patients had a skin or soft tissue infection, and 4 patients had septic arthritis. In 6 cases no focal infection was found.

At admittance 24 of the 38 patients fulfilled the SIRS-criteria [25], displaying two or more of temperature <36°C or >38°C; heart rate >90 beats per minute; respiratory rate >20 per minute; leukocyte count of >12×10^9^ L^−1^ or <4×10^9^ L^−1^. No patient had organ dysfunction at the time of experiment as judged by the result of standard blood analyses. On the day of experiment, 10 patients still met the SIRS-criteria. At the time of experiment, median platelet count was 235×10^9^ L^−1^ or (interquartile range, 160×10^9^ L^−1^–319×10^9^ L^−1^) and median neutrophil count was 7.1×10^9^ L^−1^ (5.1×10^9^ L^−1^–9.4×10^9^ L^−1^).

Nine patients were investigated within 48 hours after blood cultures had been taken, 9 were investigated between 48 and 72 hours, and another 9 were investigated within 72 to 96 hours after cultures had been drawn. Eleven patients were investigated between 96 and 120 hours after blood cultures had been obtained.

Thirteen patients, including all patients infected with enterococci, were taking, or had taken, aspirin, ibuprofen, or clopidogrel in the previous ten days.

Of the 15 controls, 9 were male and 6 were female. The mean age was 31 years (range, 23–54).

### Platelet aggregation by clinical isolates of Gram-positive bacteria

**Figure 1:**
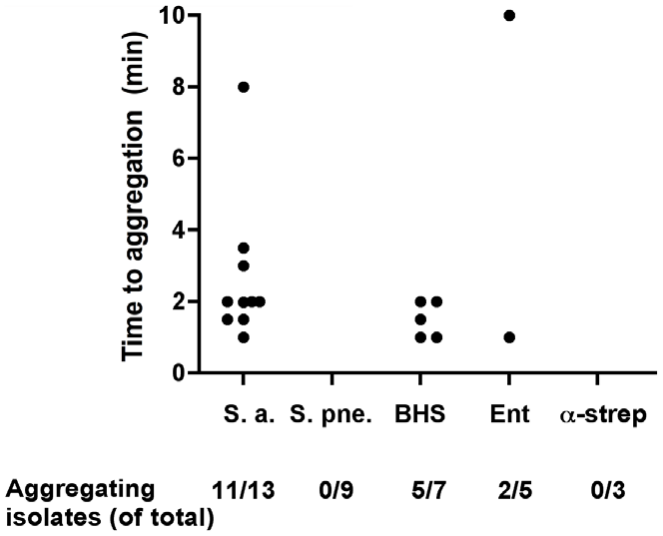
Platelet aggregation by Gram-positive pathogens. Time to aggregation of platelets in PRP from patients is shown. Filled circles depict aggregating isolates. Below the diagram are the numbers of aggregating isolates divided by the total number of isolates of the respective species presented. Abbreviations, which are used throughout the figures, are *Staphylococcus aureus* (S. a), *Streptococcus pneumoniae* (S. pne), beta-hemolytic streptococci (BHS), enterococci (Ent), and alpha-hemolytic streptococci (α-strep).

Pilot experiments using PRP from healthy donors and reference isolates of *S. aureus*, *S. pneumoniae*, GAS, and *E. faecalis*, demonstrated that all strains, with the exception of *S. pneumoniae*, caused platelet aggregation within 25 minutes (data not shown). The S. pneumoniae strain was also tested at a higher concentration (final concentration of 1.6·10^8^ bacteria/ml) but still failed to induce platelet aggregation. All isolates from the patients included in our study were tested for their ability to induce aggregation of platelets in PRP from the respective patient. Collagen was used as a positive control, and one patient (no. 14, Table 1), whose platelets did not aggregate in response to collagen, was excluded from further analysis of platelet aggregation. Eleven of 13 isolates of *S. aureus* and 5 of 7 BHS isolates induced aggregation, as did 2 of 5 enterococcal isolates. All isolates of *S. pneumoniae* (n = 10) and alpha hemolytic streptococci (n = 3) failed to induce aggregation. Time to aggregation was between 1 and 10 minutes (Fig. 1). For one aggregating *S. aureus* isolate it was not possible to determine the lag time to aggregation.

Platelets from 5 of the 13 patients treated with an antiplatelet drug responded with aggregation when stimulated with the bacteria that had been isolated from their blood. The corresponding number for platelets from patients not on antiplatelet medication was 13 out of 24. There was no significant difference using Fisher's exact test in the distribution of platelet aggregating isolates and antiplatelet treatment.

### Platelet activation

Stimulation of platelets by ADP or by certain bacteria induces activation marked by phenotypic changes and release of granules. This was monitored by analyzing platelet-rich plasma by three-color flow cytometry. Platelets were identified using a CD42 specific antibody and release of alpha granules was determined using a CD62P specific antibody. The activation status of the fibrinogen receptor (GPIIb/IIIa) was determined using PAC-1, which is an antibody specific for the active conformation of the receptor. PRP was incubated with either HEPES buffer, ADP, or the bacterium isolated from the patient. The incubation time and bacterial concentration was chosen as in an earlier study [8], and pilot experiments showed that all reference strains, except *S. pneumoniae*, induced platelet activation under these conditions (data not shown). Platelet activation was determined for platelets in PRP from healthy controls and from patients. The percentage of platelets exhibiting the activation marker CD62P correlated with the mean fluorescence intensity (MFI) of the same marker (r = 0.79, 0.86, and 0.88 with the Spearman correlation coefficient for platelets incubated with buffer only, with ADP, or with bacteria; data not shown).

**Figure 2:**
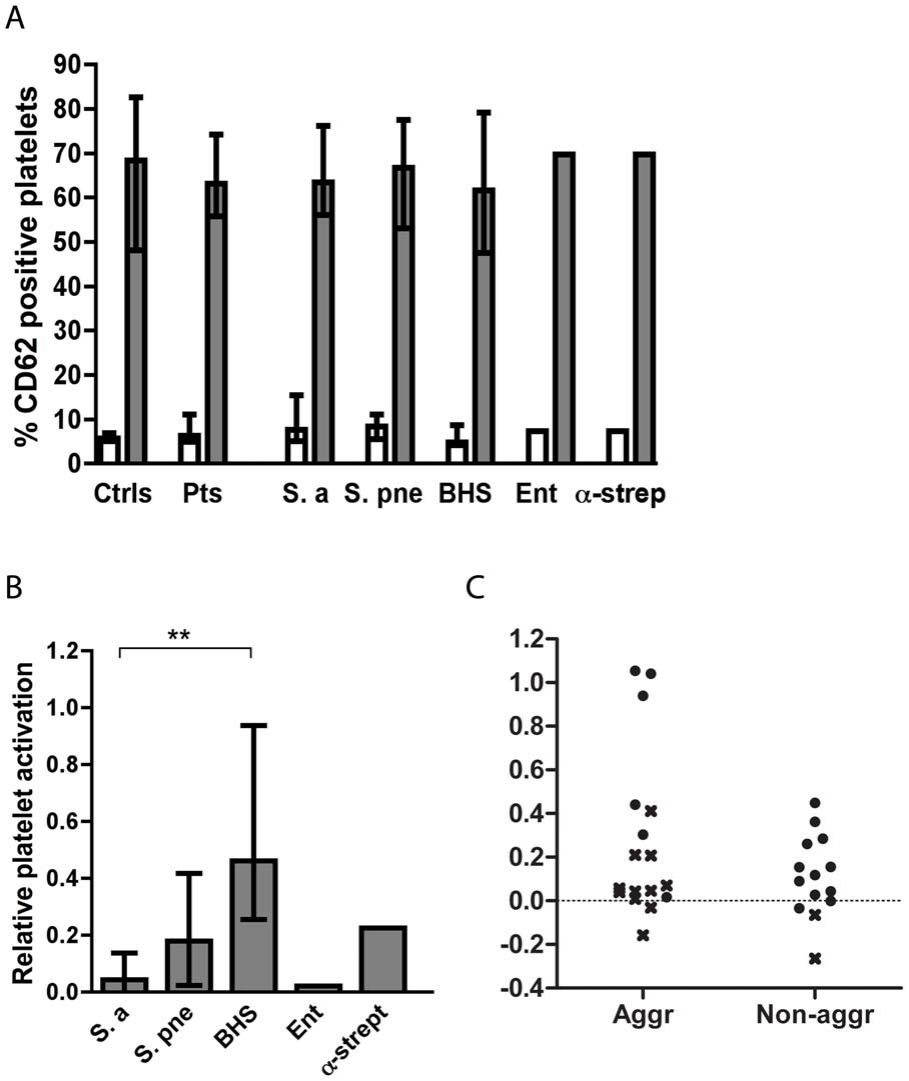
Platelet activation in healthy controls and in patients. (A) Platelet activation in healthy controls (ctrls), in all patients (pts), and in patients grouped by their respective infective organism. Platelet activation is shown as the percentage of CD 62-positive platelets in response to buffer only (open bars) or to ADP (grey bars). In (B) the increase in activated platelets in response to different bacterial isolates compared to buffer relative to the increase caused by stimulation by ADP compared to buffer is shown. Values are expressed as medians ± interquartile range. *P<0.05 after Mann-Whitney U testing. In (C) the percentage of CD 62-positive platelets in response to bacteria in patient blood is shown in two groups. To the left, patients responding with platelet aggregation to the bacterium are shown and to the right patients not responding with aggregation to the bacterium are depicted. Crosses represent *S. aureus*, while filled circles represent other bacteria. The difference between the groups is significant only if the results obtained with the *S. aureus* isolates are excluded.

The median percentage of activated platelets, as determined by CD62P-presentation, in PRP of patients was 6.3 (interquartile range, 4.9–12) as compared to 5.7 (5.2–7.0) in healthy controls. Using the Mann-Whitney U test the difference was not significant (*P* = 0.47). The corresponding numbers after stimulation with ADP were in patients 65 (56–75) and in healthy controls 68 (48–83) percent (*P* = 0.42 for a difference) (Fig. 2A). As judged by the Kruskal-Wallis test there were no statistically significant differences between platelet activation by buffer only or by ADP in patients infected by the different organisms studied (Fig. 2A). Patients no. 14, 18, 20, 30, 31, 32, and 35 were excluded from the analyses of platelet activation since platelets in PRP from these patients were either too few to be measured, had aggregated before flow cytometry or failed to respond to ADP.

Platelet activation in patient PRP by bacteria isolated from the respective patient varied depending on the organism. In Figure 2B, bacterial activation of platelets (as determined by CD62P-presentation) is shown relative to the activation induced by ADP. BHS activated platelets to a significantly higher extent than *S. aureus* (*P* = 0.0015 with the Mann-Whitney U test), but not significantly more than *S. pneumoniae* (*P* = 0.13). *S. aureus* did not activate platelets significantly more than did buffer alone (*P* = 0.07 with the Wilcoxon matched-pairs signed rank test), while *S. pneumoniae* induced significantly more activated platelets than buffer did (*P* = 0.04).

The increase in platelet activation by bacteria relative to activation by ADP was not higher among the aggregating than the non-aggregating isolates (*P* = 0.42 using the Mann-Whitney U test) (Fig. 2C). Many *S. aureus* isolates failed to induce platelet activation as judged by the FACS-analysis even though they induced aggregation as judged by turbidometry. Thus, if the isolates of *S. aureus* (depicted with crosses in Fig. 2C) were excluded, the remaining aggregating isolates induced significantly more platelet activation than did the non-aggregating isolates (*P* = 0.035).

Activation of platelets from patients and controls as determined by the percentage of platelets expressing the active conformation state of GPIIb/IIIA did not differ significantly compared to the activation determined by the percentage of platelets positive for CD62P (see Data S1). In Data S1, numerical values for platelet activation, neutrophil activation, and PNC formation are given for each patient.

### Neutrophil activation

Upon activation, neutrophils increase the expression of CD11b, which together with CD18 form the Mac-1 complex that is important for adhesion and migration [26]. We monitored the level of neutrophil activation by determining the surface expression of CD11b, measured as the MFI.

**Figure 3:**
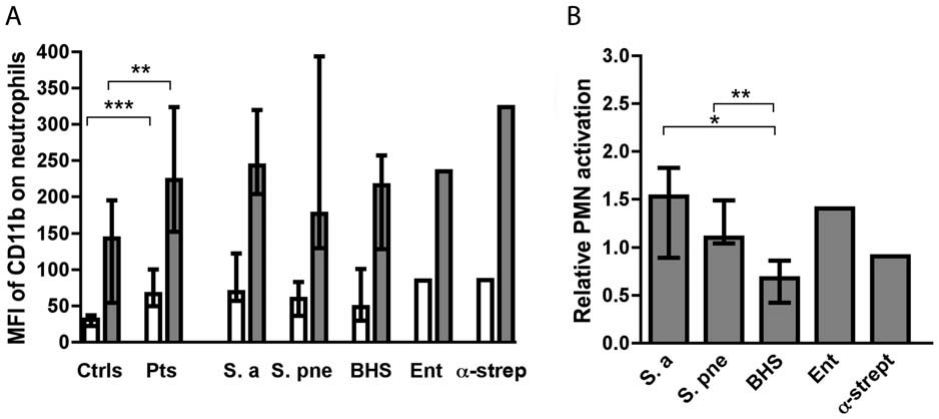
Neutrophil activation in healthy controls and in patients. (A) Neutrophil activation in healthy controls, in all of the patients, and in patients grouped by their respective infective organism. Neutrophil activation is given as the MFI of CD11b in response to buffer only (open bars) or ADP/fMLP (grey bars). Values are ± interquartile range. In (B) the increase in MFI of CD11b by different bacteria relative to the increase caused by stimulation of ADP is shown. *P<0.05, **P<0.01, ***P<0.001 after Mann-Whitney U testing.

Median MFI of CD11b on neutrophils in blood from patients was 67 (interquartile range, 50–100), which was statistically significantly higher than the level in healthy controls that was 32 (23–37) (*P*<0.0001) (Fig. 3A). Stimulation of neutrophils in blood from patients with ADP and formyl-methionyl-leucyl-phenylalanine (fMLP) in combination (ADP/fMLP) increased the MFI of CD11b to a median of 223 (153–325) and to 143 (55–196) in healthy controls (*P* = 0.008 for a difference with the Mann-Whitney U test). As judged by the Kruskal-Wallis test there were no statistically significant differences between MFI of CD11b on neutrophils from patients infected by different bacteria, either in buffer or after stimulation with ADP/fMLP (Fig. 3A). Isolates from patients no. 14, 24, 30, 31, and 35 were not included in the analyses of neutrophil activation since expression of CD11b was not determined for these patients.

Stimulation of neutrophils from patients with their respective bacterium resulted in a varied response. This is shown in Fig. 3B as the increase in activation induced by the bacteria relative to that induced by ADP and fMLP. Most bacterial isolates induced surface expression of CD11b on neutrophils to approximately the same extent as ADP/fMLP did. *S. pneumoniae* and *S. aureus* induced significantly more neutrophil activation than BHS (*P* = 0.007 and *P* = 0.045). Other differences were not significant. Two patients, one infected with *S. aureus* and one with GAS, were excluded from the latter analyses since ADP/fMLP failed to cause an increase in MFI of CD11b.

### Platelet-neutrophil complexes

The formation of complexes of neutrophils and platelets was determined as the percentage of neutrophils positive for the platelet marker CD61 which is present at the surface of resting and activated platelets.

**Figure 4:**
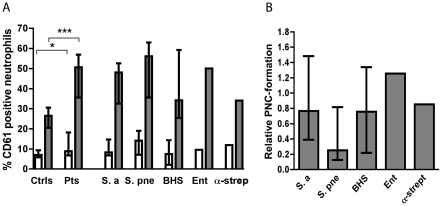
Formation of platelet-neutrophil complexes in healthy controls and in patients. (A) The percentage of PNCs is expressed as the percentage of neutrophils positive for the platelet marker CD61 in response to buffer only (open bars) or ADP/fMLP (grey bars). Values are ± interquartile range. In (B) the increase in PNCs induced by different bacterial isolates relative to the increase caused by ADP/fMLP is shown. *P<0.05 and ***P<0.001 with Mann-Whitney U test.

The median percentage of PNCs in whole blood from patients was 8.9 (interquartile range, 6.9–18.3) as compared to 7.0 (5.9–9.4) in healthy controls (*P* = 0.04 for a difference with the Mann-Whitney U test). After stimulation with ADP/fMLP the median percentage of neutrophils in complex with platelets were 51 (37–79) in patients and 27 (21–31) (*P* = 0.0005) in controls. As judged by the Kruskal-Wallis test there were no statistically significant differences between the percentages of PNCs in patients infected by different organisms (Fig. 4A). Patients no. 14, 30, 31, and 35 were not included in the analyses of PNC formation.

Stimulation of blood from patients with their respective bacterium resulted in most cases in increased PNC formation. As judged by the Kruskal-Wallis test there was no significant difference in the relative increase in PNCs induced by different bacteria (Fig. 4B). However, using the Wilcoxon's matched-pairs signed rank test, *S. pneumoniae* isolates induced significantly less PNC formation than ADP/fMLP in the respective patients (*P* = 0.002).

### Platelet and neutrophil responses to *S. aureus* in patients and healthy controls

**Figure 5:**
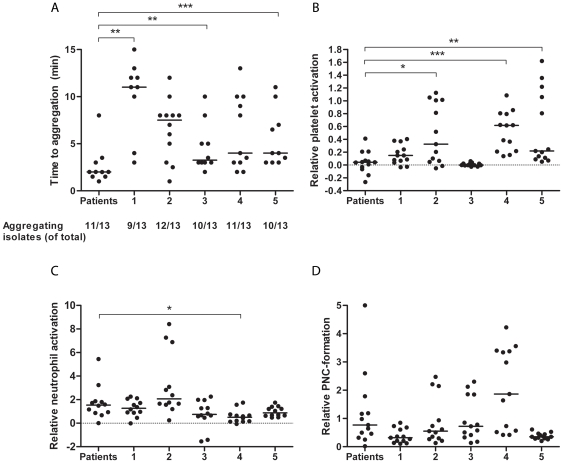
Platelet and neutrophil responses to clinical blood isolates of *S. aureus*. (A) Time to platelet aggregation in PRP by 13 *S. aureus* isolates is shown. In the left part of the figure, time to aggregation in PRP of patients with *S. aureus* bacteremia is shown. Time to aggregation in the five healthy donors (labeled 1–5) is shown in the right part of the figure. In the bottom of the figure, the number of isolates inducing aggregation of platelets is given. (B) The increase in activated platelets by the 13 *S. aureus* isolates relative to the increase caused by stimulation of ADP is given for the patients and for the five donors (1–5). (C) The increase of MFI of CD11b on neutrophils induced by the 13 *S. aureus* isolates relative to the increase caused by stimulation of ADP/fMLP is given for the patients and for the five donors. (D) The increase in PNCs induced by the 13 *S. aureus* isolates relative to the increase caused by stimulation of ADP/fMLP is given for the patients and for the five donors. One value in (D) is above five (8.3) and is depicted on the outer line of the graph. A filled circle represents each isolate. Medians are given as lines. *P<0.05, **P<0.01, ***P<0.001 after Mann-Whitney U testing.

The apparent discrepancy in the ability of the isolates of *S. aureus* in our study to cause platelet aggregation and platelet activation in PRP of patients made us investigate how these isolates affect platelets and neutrophils from 5 healthy donors. The results are summarized in Figs. 5A–D. Most isolates (10/13) aggregated platelets from 4 or all 5 donors, and 2 isolates aggregated platelet from 3 donors. Notably, the *S. aureus* isolated from patient no. 7 failed to induce aggregation in the patient as well as in all 5 healthy donors. There was a tendency for shorter time to aggregation in the patients as compared to the donors. The difference reached significance for donor 1 (*P* = 0.0051), donor 3 (*P* = 0.005), and donor 5 (*P* = 0.02), and were close to significance for donor 2 (*P* = 0.09) and donor 4 (*P* = 0.07) using the Mann-Whitney U test (Fig. 5A). One aggregating isolate was excluded from this analysis since it was not possible to determine the lag time to aggregation for this isolate in PRP from the patient.

Platelet activation by the *S. aureus* isolates expressed as the increase in the percentage of CD62P positive platelets relative to the increase induced by ADP is shown in Figure 5B. The relative increase in platelet activation, as determined by the percentage of CD62P positive platelets, by the *S. aureus* isolates was significantly higher in 3 of the 5 donors as compared to the relative increase by the respective isolate in its patient (*P*-values = 0.01, 0.0002, and 0.0015 for donors 2, 4, and 5, respectively with the Mann-Whitney U test). In donor 3, no isolate induced significant activation. For platelet activation, as determined by the percentage of platelets expressing the active conformation of GPIIb/IIIa-receptor, the relative increase in platelet activation by the *S. aureus* isolates was significantly higher in donors 1, 2, and 4 (*P*-values = 0.03, 0.007, and 0.0002) than in patients (data not shown).

Isolates of *S. aureus* induced neutrophil activation in most donors with a median activation of 1.2 (interquartile range 0.62–1.7) relative to the activation of induced by ADP/fMLP (Fig. 5C). There was a tendency toward greater neutrophil activation by *S. aureus* in the patients than in the healthy controls; however, the increase induced by the bacteria relative to that induced by ADP/fMLP was significantly higher in patients only compared to one donor (donor 4) (Fig. 5C).

PNC formation in response to *S. aureus* relative to that induced by ADP/fMLP was significantly higher in patients than in donor 1 and 2 (*P* = 0.02 and *P* = 0.02 using the Mann-Whitney U test) (Fig. 5D).

## Discussion

Despite numerous studies on bacterial platelet aggregation induced by bacteria this is the first report where bacteria have been tested in blood from the host they had infected. This is very relevant since platelet aggregation is in most cases IgG-dependent and thus donor dependent [6], [8], [27]. Earlier studies have relied on healthy platelet donors, and even among such subjects platelet aggregation in response to bacteria can be variable [8], [28]. The current study uses an *in vivo*-like setup to demonstrate that most isolates of *S. aureus*, GAS, GGS, and *E. faecalis*, can induce platelet aggregation in their host. This provides evidence that platelet aggregation by bacteria can occur during severe infections and may be an important event. We have also demonstrated that PNC formation and neutrophil activation was more abundant in baseline samples from patients than in healthy controls and that most bacterial isolates induced additional PNC formation and neutrophil activation.

Most of the *S. aureus* isolates in our study induced aggregation of platelets from their respective hosts as well as from healthy controls. Using PRP from healthy donors, a lower frequency of platelet aggregation has previously been reported for clinical isolates of *S. aureus* [11]. Whereas we observed no differences in the frequency of aggregation induced by *S. aureus* isolates between patients and controls, aggregation occurred more rapidly in patients though the differences did not always reach statistical significance. The faster aggregation could be attributable to higher antibody levels, increased platelet reactivity due to the inflammatory process in the patients, or perhaps higher levels of the acute phase protein fibrinogen which is important for several types of bacterial platelet aggregation [10], [13], [14], [15]. In the light of these findings we were surprised that the same *S. aureus* isolates failed to induce platelet activation in the patients. We therefore studied the platelet activation in response to these isolates also in healthy donors and found that platelet activation occurred in most donors. Since platelet aggregation occurred in response to the *S. aureus* isolates, we find it likely that this was preceded by platelet activation also in the patients but that our method and detection at a single fixed time-point and bacterial-platelet ratio failed to demonstrate this. Our method could however detect both streptococcal and enterococcal platelet activation in the patients and, as mentioned above, the *S. aureus* isolates induced activation in most healthy controls. Hence, we cannot formally exclude the possibility that platelet aggregation and activation may not reflect identical molecular processes in patients with bacteremia. This merits further investigations.

The lack of platelet aggregation by *S. pneumoniae* in the PRP of patients was also somewhat surprising since an earlier study had indicated that this bacterium can aggregate platelets [9]. In addition, a very recent paper also demonstrated strain dependent platelet aggregation by *S. pneumoniae* [29]. Both these reports used higher concentrations of bacteria than was used in this study. Since platelet aggregation is known to be dependent on the ratio between bacteria and platelets [8], [30], [31] the differences in bacterial concentration could perhaps explain why we did not observe platelet aggregation by *S. pneumoniae*.

Interestingly, we found no significant differences in platelet aggregation by bacteria between patients treated with an antiplatelet drug or patients without such treatment. The power of the study to detect such differences was very limited, since relatively few patients were taking antiplatelet medication and since those patients were infected with different organisms. Despite this, our findings suggest that bacteria may activate and aggregate platelets even in hosts treated with, for example, aspirin. In addition, the platelets of patients treated with aspirin responded both to collagen with aggregation and to ADP with activation (data no shown) to the same extent as platelets from the patients not treated with aspirin. Elderly patients and patients with multiple diseases which increase the risk for sepsis are more likely to receive anti-platelet drugs. Thus, the impact of such medication on the role of platelets and PNCs in sepsis should to be taken into consideration in future studies.

We also demonstrate that most bacterial isolates induce PNCs in the blood of their hosts indicating that such complexes are formed during infection. Our finding that PNCs are more prevalent in the blood of patients with Gram-positive bacteremia than in healthy controls is in agreement with previous studies that describe a higher presence of PNCs in uncomplicated septic episodes but decreased PNC-levels in sepsis complicated by organ failure, focusing on the first 24 hours of disease [20], [32], [33], [34]. None of our patients had organ failure at the time of the experiments. Most previous studies have reported elevated platelet activation in sepsis, but we did not observe this in our patients. These diverging results might reflect changes in the inflammatory state occurring during the lag time from the onset of disease to the time our experiments were carried out. We did observe that neutrophils from patients were significantly more activated than neutrophils from healthy controls. This is in agreement with Peters et al. who found that there is increased neutrophil but not platelet activation at an early stage in severe meningococcemia [22]. To promote understanding of platelet-leukocyte interactions in severe infectious disease, platelet activation and platelet-neutrophil interactions should be monitored during different stages of and time points during sepsis.

Our findings indicate that bacterial induced platelet aggregation and PNC-formation are likely to occur in infections with Gram-positive bacteria. This underlines the importance of interactions between bacteria, platelets, and leukocytes in invasive bacterial disease. A better understanding of how these cells interact, and the consequences of such interactions, may be valuable for development of novel strategies for the treatment of sepsis.

## Materials and Methods

### Ethics statement

The regional Ethical Review Board in Lund approved the study (reference number 657/2008), and written informed consent was obtained.

### Patients and controls

Patients aged 18 or above treated at the Clinic for Infectious Diseases, and patients treated in cooperation with an infectious disease specialist at other clinics at Skåne University Hospital, Lund, Sweden, with one or more blood cultures positive for a Gram-positive bacterium were considered for inclusion in the study. Patients with blood cultures taken more than five days before the day of experiment, and patients whose blood cultures grew Coagulase-negative staphylococci, diptheriod rods, or two or more bacterial species, were not considered for inclusion. The medical records for the remaining patients were reviewed, and patients with hematological malignancies and patients receiving cytostatic drugs were also not considered. Remaining patients were offered inclusion.

On the day of experiment, in conjunction with the collection of blood samples (see below under Blood samples), the patient's pulse, blood pressure, breathing frequency, oxygen saturation, and temperature were recorded. The medical records were reviewed retrospectively, and data on the current infection as well as underlying and predisposing diseases, and medication, were collected from medical records.

Fifteen healthy volunteers who had not taken antiplatelet medication in the previous ten days were recruited to the study under the same ethical approval as mentioned above.

### Bacteria and culture conditions

Bacterial isolates were collected from the accredited diagnostic laboratory for Clinical Microbiology, Skåne University Hospital, Lund, Sweden. Isolates were classified according to the standard procedures of this laboratory. *Staphylococcus aureus* (Newmann), *Streptococcus pyogenes* (AP1) [35], *Streptococcus pneumoniae* (TIGR4), and *Enterococcus faecalis* (Bef5) [8] were used as reference bacteria.

On the day of experiment, about 10 colonies of the clinical isolate taken from a blood agar plate were suspended in 10 ml of Todd Hewitt Broth (Bacto) and cultivated at 37°C with 5% CO_2_ to OD_620_ of 0.5, harvested by centrifugation at 2000 g for 10 minutes, washed once in phosphate buffered saline (PBS), and suspended in the same buffer. Bacterial concentration was adjusted by spectrophotometry at 620 nm using a standard curve obtained for a group G streptococcal strain (G148) to correspond to 2•10^9^ bacteria per ml. After suspension, the bacteria were kept on ice until experiments were carried out.

### Blood samples

After preparing bacterial suspensions, blood samples were collected from the patient from whom the bacterium had been isolated. Determination of white blood cell count, absolute neutrophil count, platelet count, hemoglobin, creatinine, bilirubin, prothrombin time/international normalized ratio (PT/INR), and activated partial thromboplastin time (APTT) were performed by the accredited routine laboratory of Clinical Chemistry and Pharmacology, Skåne University Hospital, Lund, Sweden.

Blood for analysis in our research laboratory was collected at the same time in citrated plastic tubes (BD, Plymouth, UK). For aggregometry, citrated blood was centrifuged at 160 g for 10 minutes to produce an upper platelet rich plasma (PRP), which was removed. Subsequent centrifugation at 2000 g for 10 minutes produced an upper platelet poor plasma (PPP).

### Aggregometry

A dual channel platelet aggregometer (ChronoLog model 490) was used to assess platelet aggregation by turbidometry. PPP was used in the reference cell to establish a baseline of 100% transmission. PRP was used in the test cell and the change in transmission over time was an indication of the platelet aggregation. Twenty µl of bacterial suspension (2•10^9^ cfu/ml) was added to 450 µL of PRP in the test well and the platelet response was monitored for a maximum of 25 minutes. Two µl of soluble collagen I (1 mg/ml) (Triolab, Sweden) was added to 450 µl of PRP as a positive control. Results were analyzed using the Aggrolink, version 5.2.1 software.

### Flow cytometry

For analysis of platelet activation by the bacteria, 20 µl of PRP was incubated for 25 minutes at room temperature with 40 µl of HEPES buffer pH 7.4, either in the presence or absence of washed bacteria (approximately 1.2•10^7^ bacteria) or in the presence of 5 µM adenosine diphosphate (ADP) (Sigma-Aldrich, Steinheim, Germany). After 25 minutes, 5 µL each of three fluorochrome-conjugated antibodies (CD42a-PerCP, CD62P-PE, and PAC-1FITC) (all from BD Biosciences, San Jose, CA US) was added and after 10 minutes the incubation was stopped by addition of 600 µL of 0.5% formaldehyde in ice cold PBS. Samples were analyzed using a FACSCalibur flow cytometer in logarithmic mode with a gate setting for the CD42a positive platelet population. 30,000 cells were acquired and analyzed using Cell Quest Software (Becton Dickinson).

For analysis of neutrophil activation and PNC formation in response to the bacterium that grew in the patient's blood, 20 µl of citrated whole blood from the patient was incubated for 10 minutes at room temperature with 40 µl of the same buffer as above, either in the presence or absence of bacteria (approximately 1.2•10^7^ washed bacteria) or in the presence of 5 µM ADP and 1 µM fMLP (Sigma-Aldrich). After 10 minutes, 5 µL each of three fluorochrome conjugated antibodies (CD45-FITC from DAKO, and CD61-PE and CD11b-PeCy5 from BD Biosciences) was added and after 10 minutes the incubation was stopped, and erythrocytes were lysed, using the Uti-lyse kit (DAKO, Glostrup, Denmark). Samples were analyzed as above but in linear mode with a gate setting for the part of the population that had a forward and side scatter characteristic of neutrophils and was CD45-FITC positive. 10,000 cells were acquired and analyzed using Cell Quest software (Becton Dickinson). CD61-positive neutrophils were considered to be PNCs. The percentage of the total neutrophil population positive for CD61 (that is, associated with platelets) is given as the % PNCs. Neutrophil activation was expressed as the Mean Fluorescence Intensity (MFI) of CD11b-PeCy5 of the neutrophil population.

### Statistical analysis

Data are expressed, if nothing else is stated, as medians ± the interquartile range.

Relative platelet activation, neutrophil activation, and PNC formation by bacteria was calculated as the value of the respective marker (CD62P on platelets, MFI of CD11b on neutrophils, and CD61 on neutrophils) induced by bacteria minus the value in the unstimulated sample from the same donor. This value was divided by the value of the same marker induced by ADP and ADP/fMLP, respectively, minus the value in the unstimulated sample.

Statistical analyses were performed with GraphPad Prism, version 5.03 for Windows (GraphPad Software, San Diego, CA, US). Fisher's exact test was performed using an interactive calculation tool available from http://www.quantpsy.org. P-values<0.05 were considered statistically significant.

## Supporting Information

Data S1 Interactions between bacteria, platelets, and neutrophils in patients with Gram-positive bacteremic infection.
(XLS)

## References

[1] Angus DC, Linde-Zwirble WT, Lidicker J, Clermont G, Carcillo J (2001). Epidemiology of severe sepsis in the United States: analysis of incidence, outcome, and associated costs of care.. Crit Care Med.

[2] Annane D, Bellissant E, Cavaillon JM (2005). Septic shock.. Lancet.

[3] Russell JA (2006). Management of sepsis.. N Engl J Med.

[4] Hotchkiss RS, Karl IE (2003). The pathophysiology and treatment of sepsis.. N Engl J Med.

[5] Semple JW, Freedman J (2010). Platelets and innate immunity.. Cell Mol Life Sci.

[6] Fitzgerald JR, Foster TJ, Cox D (2006). The interaction of bacterial pathogens with platelets.. Nat Rev Microbiol.

[7] Kerrigan SW, Cox D (2010). Platelet-bacterial interactions.. Cell Mol Life Sci.

[8] Rasmussen M, Johansson D, Söbirk SK, Mörgelin M, Shannon O (2010). Clinical isolates of *Enterococcus faecalis* aggregate human platelets.. Microbes Infect.

[9] Zimmerman TS, Spiegelberg HL (1975). *Pneumococcus*-induced serotonin release from human platelets. Identification of the participating plasma/serum factor as immunoglobulin.. J Clin Invest.

[10] Pietrocola G, Schubert A, Visai L, Torti M, Fitzgerald JR (2005). FbsA, a fibrinogen-binding protein from *Streptococcus agalactiae*, mediates platelet aggregation.. Blood.

[11] Rindi S, Cicalini S, Pietrocola G, Venditti M, Festa A (2006). Antibody response in patients with endocarditis caused by *Staphylococcus aureus*.. Eur J Clin Invest.

[12] Sullam PM, Jarvis GA, Valone FH (1988). Role of immunoglobulin G in platelet aggregation by viridans group streptococci.. Infect Immun.

[13] Sjöbring U, Ringdahl U, Ruggeri ZM (2002). Induction of platelet thrombi by bacteria and antibodies.. Blood.

[14] Loughman A, Fitzgerald JR, Brennan MP, Higgins J, Downer R (2005). Roles for fibrinogen, immunoglobulin and complement in platelet activation promoted by *Staphylococcus aureus* clumping factor A.. Mol Microbiol.

[15] Fitzgerald JR, Loughman A, Keane F, Brennan M, Knobel M (2006). Fibronectin-binding proteins of *Staphylococcus aureus* mediate activation of human platelets via fibrinogen and fibronectin bridges to integrin GPIIb/IIIa and IgG binding to the FcgammaRIIa receptor.. Mol Microbiol.

[16] Gafter-Gvili A, Mansur N, Bivas A, Zemer-Wassercug N, Bishara J (2011). Thrombocytopenia in *Staphylococcus aureus* bacteremia: risk factors and prognostic importance.. Mayo Clin Proc.

[17] Brown KK, Henson PM, Maclouf J, Moyle M, Ely JA (1998). Neutrophil-platelet adhesion: relative roles of platelet P-selectin and neutrophi beta 2 (DC18) integrins.. Am J Respir Cell Mol Biol.

[18] Evangelista V, Manarini S, Rotondo S, Martelli N, Polischuk R (1996). Platelet/polymorphonuclear leukocyte interaction in dynamic conditions: evidence of adhesion cascade and cross talk between P-selectin and the beta 2 integrin CD11b/CD18.. Blood.

[19] Kirschenbaum LA, Adler D, Astiz ME, Barua RS, Saha D (2002). Mechanisms of platelet-neutrophil interactions and effects on cell filtration in septic shock.. Shock.

[20] Gawaz M, Fateh-Moghadam S, Pilz G, Gurland HJ, Werdan K (1995). Platelet activation and interaction with leucocytes in patients with sepsis or multiple organ failure.. Eur J Clin Invest.

[21] Clark SR, Ma AC, Tavener SA, McDonald B, Goodarzi Z (2007). Platelet TLR4 activates neutrophil extracellular traps to ensnare bacteria in septic blood.. Nat Med.

[22] Peters MJ, Heyderman RS, Faust S, Dixon GL, Inwald DP (2003). Severe meningococcal disease is characterized by early neutrophil but not platelet activation and increased formation and consumption of platelet-neutrophil complexes.. J Leukoc Biol.

[23] Papapanagiotou D, Nicu EA, Bizzarro S, Gerdes VE, Meijers JC (2009). Periodontitis is associated with platelet activation.. Atherosclerosis.

[24] Brandt CM, Spellerberg B (2009). Human infections due to *Streptococcus dysgalactiae subspecies equisimilis*.. Clin Infect Dis.

[25] Bone RC, Balk RA, Cerra FB, Dellinger RP, Fein AM (1992). Definitions for sepsis and organ failure and guidelines for the use of innovative therapies in sepsis. The ACCP/SCCM Consensus Conference Committee. American College of Chest Physicians/Society of Critical Care Medicine.. Chest.

[26] Solovjov DA, Pluskota E, Plow EF (2005). Distinct roles for the alpha and beta subunits in the functions of integrin alphaMbeta2.. J Biol Chem.

[27] Miajlovic H, Loughman A, Brennan M, Cox D, Foster TJ (2007). Both complement- and fibrinogen-dependent mechanisms contribute to platelet aggregation mediated by *Staphylococcus aureus* clumping factor B.. Infect Immun.

[28] McNicol A, Zhu R, Pesun R, Pampolina C, Jackson EC (2006). A role for immunoglobulin G in donor-specific *Streptococcus sanguis*-induced platelet aggregation.. Thromb Haemost.

[29] Keane C, Tilley D, Cunningham A, Smolenski A, Kadioglu A (2010). Invasive *Streptococcus pneumoniae* trigger platelet activation via Toll-like receptor 2.. J Thromb Haemost.

[30] Bayer AS, Sullam PM, Ramos M, Li C, Cheung AL (1995). *Staphylococcus aureus* induces platelet aggregation via a fibrinogen-dependent mechanism which is independent of principal platelet glycoprotein IIb/IIIa fibrinogen-binding domains.. Infect Immun.

[31] Kurpiewski GE, Forrester LJ, Campbell BJ, Barrett JT (1983). Platelet aggregation by *Streptococcus pyogenes*.. Infect Immun.

[32] Gawaz M, Dickfeld T, Bogner C, Fateh-Moghadam S, Neumann FJ (1997). Platelet function in septic multiple organ dysfunction syndrome.. Intensive Care Med.

[33] Kirschenbaum LA, Aziz M, Astiz ME, Saha DC, Rackow EC (2000). Influence of rheologic changes and platelet-neutrophil interactions on cell filtration in sepsis.. Am J Respir Crit Care Med.

[34] Mavrommatis AC, Theodoridis T, Orfanidou A, Roussos C, Christopoulou-Kokkinou V (2000). Coagulation system and platelets are fully activated in uncomplicated sepsis.. Crit Care Med.

[35] Åkesson P, Cooney J, Kishimoto F, Björck L (1990). Protein H–a novel IgG binding bacterial protein.. Mol Immunol.

